# Closed-Loop Gangrene Post Banded Roux-en-Y Gastric Bypass (B-RYGB)

**DOI:** 10.7759/cureus.105603

**Published:** 2026-03-21

**Authors:** Mansour Alkhurmudi, Abdullah S Alzaharani, Feras Alsannaa, Khalid N Alsowaina, Dhuha A Abukhamsin

**Affiliations:** 1 General Surgery, Prince Sultan Military Medical City, Riyadh, SAU; 2 Trauma and Acute Care Surgery, Prince Sultan Military Medical City, Riyadh, SAU; 3 Minimally Invasive, Upper Gastrointestinal, and Bariatric Surgery, Prince Sultan Military Medical City, Riyadh, SAU

**Keywords:** banded gastric bypass, internal herniation, minimizer, silastic band, small bowel ischemia

## Abstract

Although laparoscopic banded Roux-en-Y gastric bypass (B-RYGB) has been adopted in bariatric surgery to enhance long-term weight loss outcomes and reduce pouch dilation, cranial slippage (herniation) of the alimentary limb through a non-slipped MiniMizer gastric ring (Bariatric Solutions International GmbH, Stein am Rhein, Switzerland) following B-RYGB, leading to small bowel ischemia, represents a rare but serious complication.

We present a case of a 35-year-old male who had undergone multiple bariatric procedures to manage obesity due to failure to lose weight. The patient presented to our emergency department in December 2024 with features of an acute surgical abdomen, eight months after undergoing laparoscopic B-RYGB using a MiniMizer gastric ring.

This case report contributes to the growing body of literature on complications associated with B-RYGB, particularly those related to the MiniMizer gastric ring.

## Introduction

The prevalence of obesity is rising [1], resulting in an increase in the number of bariatric surgery procedures performed to manage this disease and its associated comorbidities.

Failure to lose weight (insufficient weight loss or weight regain) after bariatric surgery ranges between 20% and 30% [2] and is known to be one of the most common causes for revisional surgeries [3].

New surgical techniques have been introduced and gained interest in reducing the number of patients presenting with failure to lose weight; these include narrowing the pouch, lengthening the biliopancreatic limb, and placement of a non-adjustable band [4]. Each procedure varies in weight loss results, comorbidity resolution, and complications.

Although band placement has some advantages, it has been reported that it has the same complications as the laparoscopic adjustable gastric banding (LAGB), which include food intolerance, band erosion, and slippage (migration) [5].

This case of small bowel ischemia caused by band placement during Roux-en-Y gastric bypass (RYGB) highlights the importance of long-term monitoring of banded gastric bypass procedures to evaluate their complications, weight regain, and the need for reoperation, compared to those observed with LAGB.

## Case presentation

The patient was a 35-year-old male with no significant past medical history. He had a surgical history notable for a laparoscopic mini-gastric bypass 12 years prior to his presentation, converted to a laparoscopic banded Roux-en-Y gastric bypass (B-RYGB) using MiniMizer gastric ring (Bariatric Solutions International GmbH, Stein am Rhein, Switzerland) in April 2024 due to failure to lose weight (body mass index = 47 kg/m^2^).

The patient presented to our emergency department in December 2024, complaining of colicky generalized abdominal pain mainly in the epigastric area for three days associated with recurrent vomiting of black foul-smelling content, and food intolerance.

His vital signs were normal during our initial assessment (heart rate of 85, blood pressure of 127/87, respiratory rate of 20, oxygen saturation of 99% on room air, and he was afebrile). His last body mass index was 39 kg/m^2^. He was looking dehydrated and in pain. Abdominal examination revealed only mild epigastric tenderness with no guarding or rigidity.

Laboratory investigations were notable only for elevated inflammatory markers (Table 1). The serum lactate level, basic metabolic panel, and liver function tests were within normal limits.

**Table 1:** Laboratory investigations.

Laboratory investigations	Result	Reference range
White blood cell count	15.8 10^9/L	4.00 - 11.00 10^9/L
C-reactive protein	201 mg/L	0.00 - 6.00 mg/L

**Figure 1:**
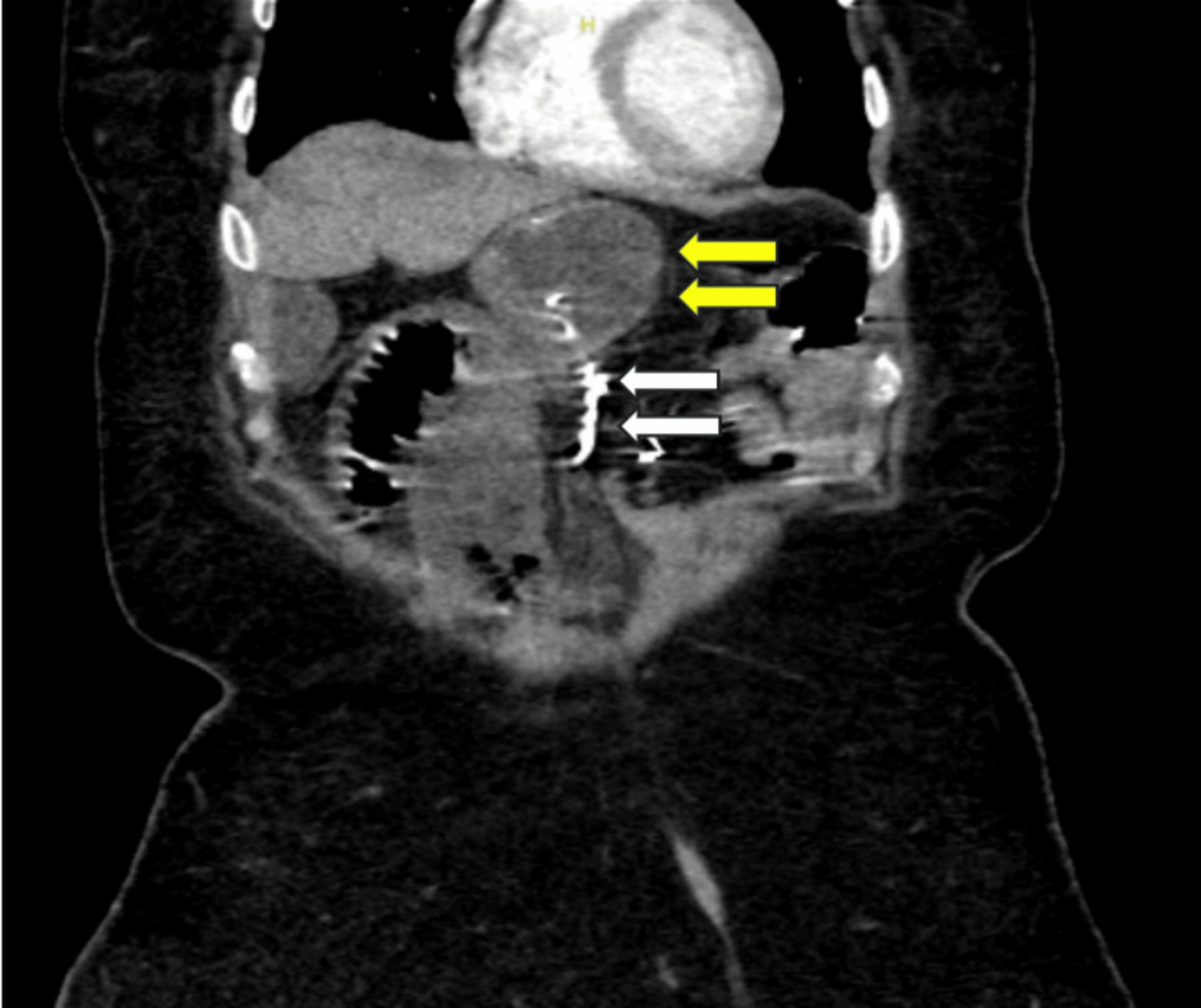
CT of the abdomen with white arrows marking the MiniMizer and yellow arrows showing a segment of an obstructed dilated small bowel loop with decreased enhancement and congested mesentery.

Computed tomography (CT) of the abdomen with intravenous (IV) contrast revealed bowel loops volvulus versus severe adhesions at the level of the gastrojejunostomy/gastric pouch with a long segment of obstructed dilated small bowel loop reaching up to 4.9 cm that showed decreased enhancement and congested mesentery with a mild amount of free fluid. The constellation of these findings was in keeping with closed-loop obstruction secondary to volvulus versus adhesions complicated by small bowel ischemia; however, there was no pneumatosis intestinalis, mesenteric venous gas, or portal venous gas. No free air to suggest perforation was noted (Figure 1).

Management

**Figure 2:**
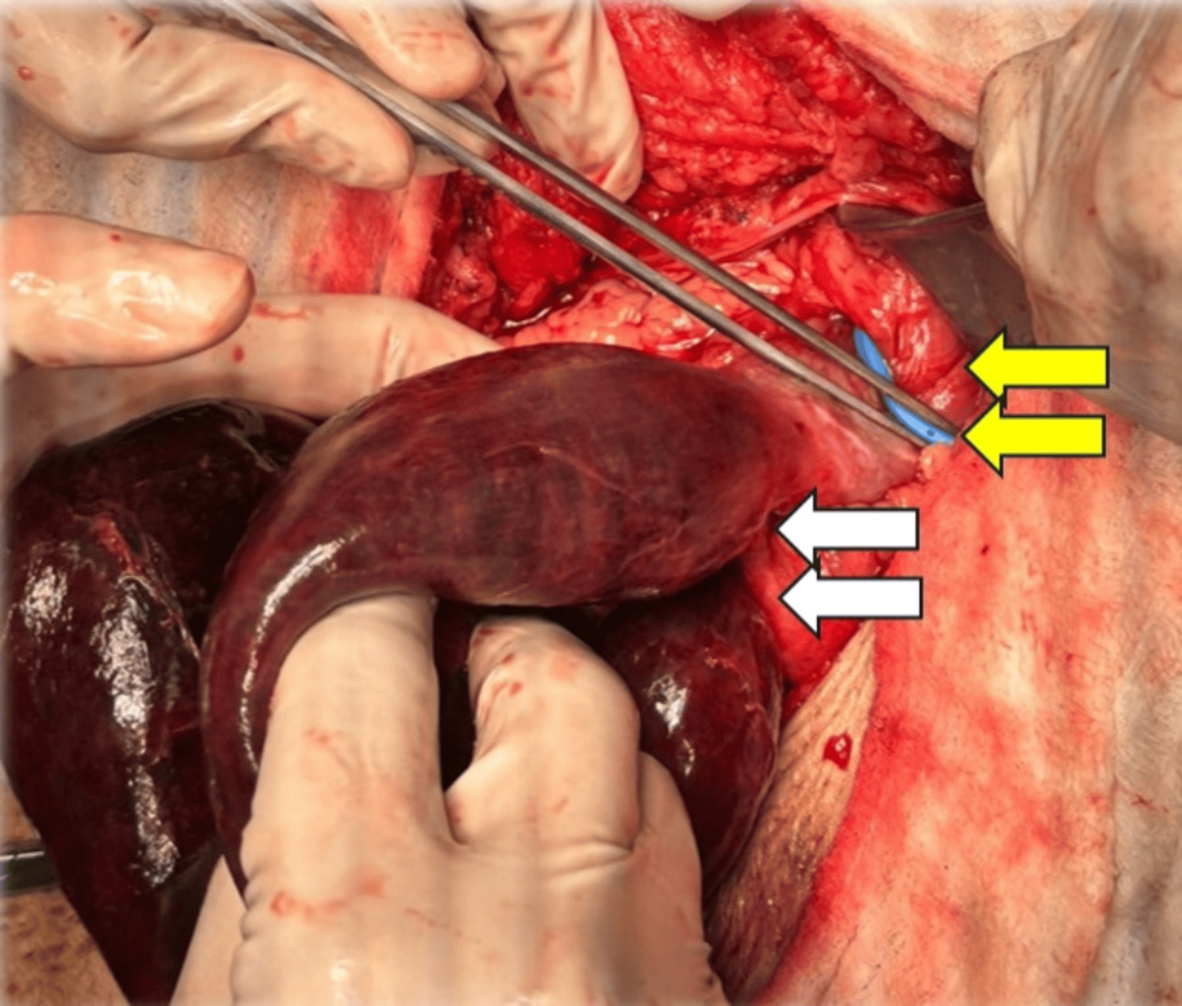
Intraoperative findings with yellow arrows marking the MiniMizer and white arrows showing the ischemic part of the small bowel.

Given the concern for a closed-loop bowel obstruction and the risk of bowel strangulation/ischemia, the patient was taken to the operating room for an emergency laparoscopic exploration. Intraoperative finding was dilated small bowel with retrograde herniation of the alimentary limb through a fixed MiniMizer gastric ring, causing closed-loop obstruction with ischemic small bowel about 45 cm in length (Figure 2).

Due to the difficulty in handling the dilated small bowel loops and to allow better evaluation and measurement of the involved segments of the Roux-en-Y bypass limbs, the decision was made to convert the procedure to a midline laparotomy. The MiniMizer gastric ring was removed, and the ischemic segment of the alimentary limb was resected. A side-to-side anastomosis was then performed using a stapling device. A leak test with methylene blue was performed and was negative. The final Roux-en-Y bypass limb measurements were as follows: alimentary limb of 50 cm, biliopancreatic limb of 100 cm, and common channel of 230 cm. Both the mesenteric and Petersen’s defects were closed. Two drains were inserted (one in the pelvis and the other near the anastomosis site), and the abdomen was closed in layers.

Outcome and follow-up

The patient had an uneventful postoperative course and was started on oral intake on postoperative day three according to our bariatric and metabolic surgery unit protocol (clear fluids for one week, full liquids during the second week, a soft diet in the third week, followed by a regular diet as per the dietitian’s instructions). He was discharged home one week after surgery with good oral intake and after removal of all surgical drains. The patient was subsequently followed up in the outpatient clinic at two weeks, one month, three months, six months, and one year. He was tolerating oral intake well and denied any symptoms of dumping syndrome. His last recorded BMI was 27 kg/m².

## Discussion

Weight loss failure can be attributed to many factors, including large gastric pouch size, gradual increase in the pouch volume, behavioral factors, and non-adherence to dietary instructions.

One effective surgical technique to reduce the incidence of failure to lose weight caused by the aforementioned factors is banding the gastric pouch with a silastic or MiniMizer ring. This additional restrictive method serves to prevent both pouch dilatation and rapid emptying of the pouch, thereby decreasing the likelihood of postoperative dumping syndrome symptoms [6].

Although band placement has some advantages, it has a complication rate of 7%, which includes stenosis at the gastrojejunostomy anastomosis site leading to food intolerance (2.8%), band erosion (0-7.7%), and slippage/migration (1.5-7.33%) [5,7-9].

In our case, the small bowel herniated retrogradely through the band, resulting in closed-loop ischemia. Several factors may contribute to this complication, including reduced mesenteric fat following weight loss, dysmotility or reverse peristalsis over a fixed point (e.g., the MiniMizer ring) due to ectopic pacemaker cells [10,11], and failure to adhere to dietary instructions, such as consuming large portions or hard foods [12]. Similar cases associated with significant morbidity have previously been reported. Mousli et al. [12] described two cases: one presenting with mechanical ileus caused by cranial herniation of the alimentary limb without slippage of the MiniMizer ring, and another involving mesenteric ischemia following ventral migration of the MiniMizer ring, with herniation of the alimentary limb and its mesentery through the ring leading to mesenteric torsion. Additionally, Unadkat et al. [13] reported a series of three cases following B-RYGB in which patients developed small bowel ischemia due to herniation of the alimentary limb between the gastric pouch and the ring.

In our literature review, we found that studies comparing B-RYGB to the standard RYGB show a statistically significant difference in excess weight loss and reduced weight regain [14,15]; however, the difference is only a few points of BMI or kilograms. Excess weight loss with B-RYGB would be 5% greater than that with the non-banded RYGB (approximately a one-point difference in body mass index) [16]. Also, we noticed that in the long-term follow-up (after 10 years), the LAGB complication rate was as high as 47%, with around 68% of patients undergoing reoperation due to complications or weight regain [17,18], and less than 40% of patients had a functioning band [19,20].

This raises the question of whether B-RYGB will have similar outcomes, leading to a new era of adverse events in the future.

## Conclusions

The development of this serious complication in our patient, i.e., small bowel ischemia related to band placement during RYGB, emphasizes the need for rigorous long-term follow-up of the B-RYGB procedure to determine whether its long-term outcomes, including complication rates, weight regain, and the need for reoperation, mirror those observed with laparoscopic adjustable gastric banding.

Early identification through a high level of suspicion and radiological investigations, along with surgical exploration and removal of the band, can help reduce patient morbidity resulting from its complications.
